# Development and Characterization of Near-Isogenic Lines Derived from Synthetic Wheat Revealing the 2 kb Insertion in the *PPD-D1* Gene Responsible for Heading Delay and Grain Number Improvement

**DOI:** 10.3390/ijms241310834

**Published:** 2023-06-29

**Authors:** Shunzong Ning, Shengke Li, Kai Xu, Dongmei Liu, Li Ma, Chunfang Ma, Ming Hao, Lianquan Zhang, Wenjie Chen, Bo Zhang, Yun Jiang, Lin Huang, Xuejiao Chen, Bo Jiang, Zhongwei Yuan, Dengcai Liu

**Affiliations:** 1 State Key Laboratory of Crop Gene Exploration and Utilization in Southwest China, Sichuan Agricultural University, Chengdu 611130, Chinalianquan@sicau.edu.cn (L.Z.); dcliu7@sicau.edu.cn (D.L.); 2 Triticeae Research Institute, Sichuan Agricultural University, Chengdu 611130, Chinahaomingluo@sicau.edu.cn (M.H.); lhuang@sicau.edu.cn (L.H.); jiangbo1988@sicau.edu.cn (B.J.); 13918@sicau.edu.cn (Z.Y.); 3 Key Laboratory of Adaptation and Evolution of Plateau Biota, Northwest Institute of Plateau Biology, Chinese Academy of Sciences, Xining 810008, China; wjchen@nwipb.cas.cn (W.C.); zhangbo@nwipb.cas.cn (B.Z.); 4 Biotechnology and Nuclear Technology Research Institute, Sichuan Academy of Agricultural Sciences, Chengdu 610061, China; m13438880787@163.com

**Keywords:** synthetic wheat, *photoperiod-1*, number of days to heading, grain number per spike, spike length, spikelet number per spike

## Abstract

Spikelet number and grain number per spike are two crucial and correlated traits for grain yield in wheat. *Photoperiod-1 (Ppd-1*) is a key regulator of inflorescence architecture and spikelet formation in wheat. In this study, near-isogenic lines derived from the cross of a synthetic hexaploid wheat and commercial cultivars generated by double top-cross and two-phase selection were evaluated for the number of days to heading and other agronomic traits. The results showed that heading time segregation was conferred by a single incomplete dominant gene *PPD-D1*, and the 2 kb insertion in the promoter region was responsible for the delay in heading. Meanwhile, slightly delayed heading plants and later heading plants obviously have advantages in grain number and spikelet number of the main spike compared with early heading plants. Utilization of *PPD-D1* photoperiod sensitivity phenotype as a potential means to increase wheat yield potential.

## 1. Introduction

As a widely cultivated crop in the world, bread wheat (*Triticum aestivum* L., AABBDD, 2n = 6x = 42) is a crucial source of calories, accounting for 20% of calories for human consumption and feeding more than 35% of the world’s population [1]. Increasing crop production is necessary to feed the world’s expanding population, and crop breeders often utilize genetic variations to improve crop yield and quality [2]. The wheat spike number per plant, spikelet number per spike, and grain number per spike are three crucial and correlated traits for grain yield in wheat. The number of fertile spikelets per spike is a determinant of the final grain number per spike at harvest, so increasing spikelet number could be an effective strategy for increasing wheat yield [3,4,5].

Bread wheat originated from allopolyploidization between the tetraploid (*T. turgidum*, AABB, 2n = 4x = 28) and the diploid (*Aegilops tauschii*, DD, 2n = 2x = 28) approximately 10,000 years ago in the Fertile Crescent. Since that time [6,7,8], intensive selection by early farmers and deliberate breeding have resulted in adaptation to a wider range of environments than occupied by any other crop species [9]. Consequently, genetic diversity is substantially reduced compared with ancestral populations, indicating a major diversity bottleneck in the transition to cultivated lines [10]. Synthetic hexaploid wheat (SHW) is an artificially created hexaploid wheat and a useful genetic resource that should be utilized to transfer needed genes from tetraploid or diploid donors, including wild species, to improve the performance of common wheat [11]. SHW has been confirmed to have better performance under biotic and abiotic stresses, as well as better yield potential, such as high grain weight and spikelet number and larger kernels and spikes [12,13,14].

Bread wheat is cultivated in virtually all countries where the photoperiod varies dramatically and continually, suggesting that there may be corresponding variations in photoperiod response among wheat varieties adapted to different environments [15]. *Photoperiod-1* (*Ppd-1*) is a key regulator of inflorescence architecture and spikelet formation in wheat by regulating the expression of *FLOWERING LOCUS T* (*FT*) [16]. It is a homeo-allelic series of loci in the 2A, 2B, and 2D chromosomes’ short arms [17,18]: *PPD-A1*, *PPD-B1,* and *PPD-D*1, respectively [19]. Moreover, while the wild-type is associated with a photoperiod-sensitive phenotype (*ppd-1b*), insensitivity allele (*Ppd-1a*) mutations are associated with a photoperiod-insensitive phenotype [20,21]. Furthermore, *Ppd-1a* reduced the number of spikelets per spike [22,23,24] by accelerating the spikelet initiation rate [25]. Their effect on time to anthesis has been extensively characterized [21,24,26,27], and reports generally agree that *Ppd-D1a* has the largest magnitude of the effect [20,21,28,29,30,31]. Lengthening the preanthesis period of stem elongation by altering photoperiod sensitivity has been suggested as a potential means to increase the number of fertile florets at anthesis to increase wheat yield potential [30].

To date, five polymorphic sites (two in the promoter region and one in the first intron, the other two polymorphisms in exons 7 and 8) have been detected in the *PPD-D1* gene, and only a candidate mutation of the 2 kb deletion in the promoter region was associated with photoperiod insensitivity [32]. Then, a series of molecular markers were developed for detecting sequence variations in *Ppd-D1* [15,32].

Although the potential of SHW for wheat improvement has been recognized for a long time, the contribution of SHW to elite commercial varieties remains relatively minor [33,34,35]. In the current study, four advanced derivatives (F_6_) with different heading dates generated from synthetic wheat were used to identify each *PPD-D1* allele responsible for phenotypic variations by population and marker analysis. The objectives of this study were (1) to evaluate the breeding potential of the derivatives and (2) to ascertain the gene(s) responsible for the delay of heading.

## 2. Results

### 2.1. Heading Date Analysis

**Figure 1:**
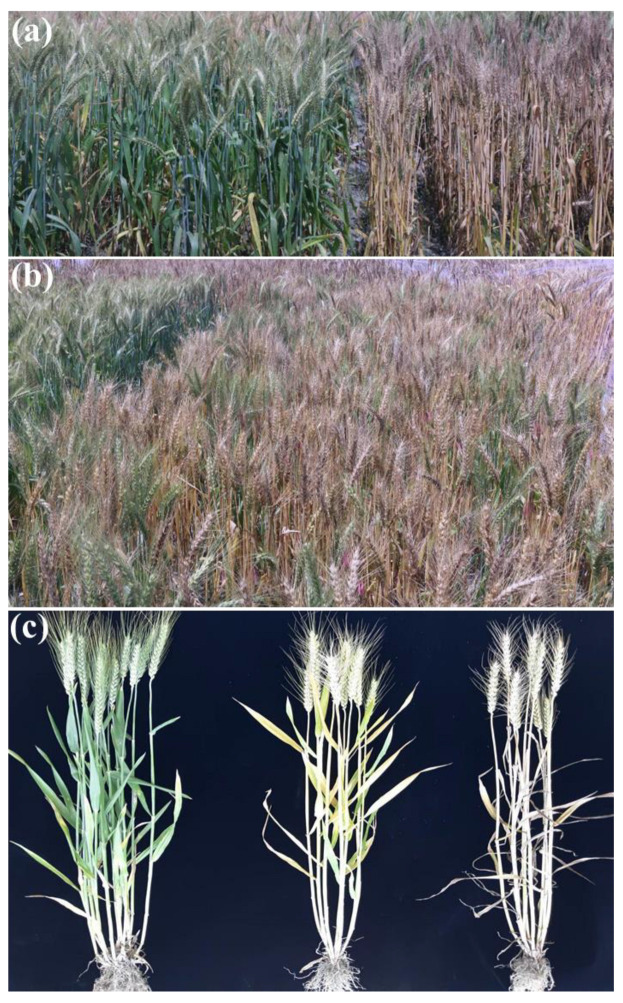
Pictures of plants in the field. (**a**) Progenies of WJN3161 (**left**) and WJN3201 (**right**); (**b**) progenies from WJN3141; (**c**) Later heading plant (**left**), slightly delayed heading plant (**middle**) and early heading plant (**right**).

There were significant differences in the heading dates of the derivatives and synthetic hexaploidy wheat Syn-SAU-24. Syn-SAU-24 had a later heading date, with 178 days from sowing to heading date. Progenies from WJN3201 and WJN3161 were consistently early heading date (approximately 131 days to heading) and later heading date (approximately 161 days to heading), respectively (Figure 1a). Due to visible differences in the heading dates of progenies from WJN3141 and WJN3151 (Figure 1b,c), two F_7_ segregating populations and their F_7:8_ populations for genetic analysis of the heading date were obtained.

**Figure 2:**
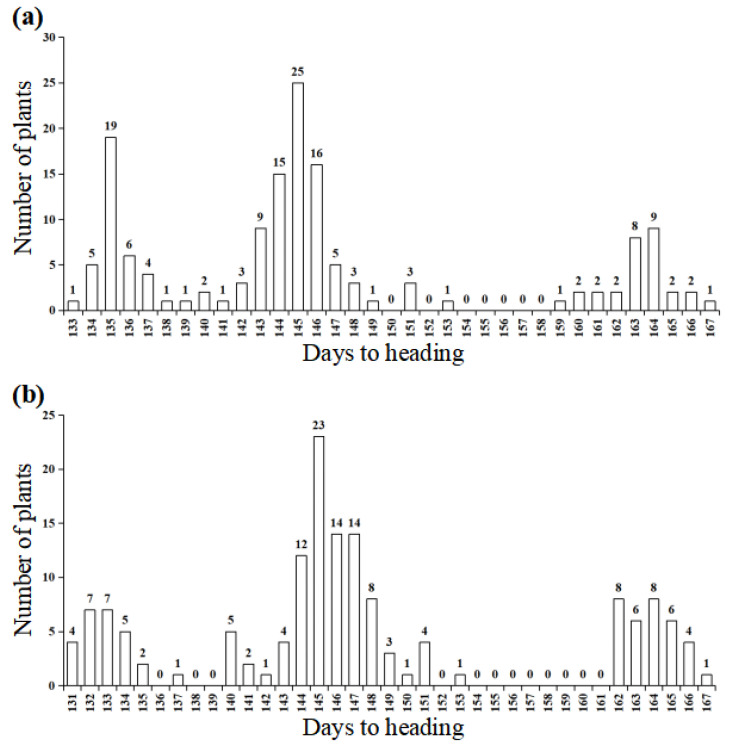
Frequency distribution of days to heading in the segregation population from WJN3141 (**a**) and WJN3151 (**b**).

Based on the heading date analysis of the F_7_ and F_7:8_ populations, heading date segregation was conferred by a single incomplete dominant gene (Table 1). The segregation populations from WJN3141 segregated in 42 early (133 to 143 days), 79 slightly delayed (142 to 153 days), and 29 later (159 to 166 days), fitting a 1: 2: 1 ratio (χ^2^_1:2:1_ = 2.680, *p* = 0.262) (Table 1, Figure 2a). The segregation populations from WJN3151 segregated in 31 early (131 to 141 days), 87 slightly delayed (137 to 153 days), and 33 later (162 to 167 days), fitting a 1: 2: 1 ratio (χ^2^_1:2:1_ = 3.556, *p* = 0.169) (Table 1, Figure 2b). Overall, the average number of days to heading was 135.9 days in early heading plants, 145 days in slightly delayed heading plants, and 162.7 days in later heading plants (Table 2). This indicates that heading date segregation was conferred by a single incomplete dominant gene.

### 2.2. Correlations of Heading Date and Agronomic Traits

All individual plants of the two F_7_ segregation populations were evaluated for plant height, number of tillers, main spike length, spikelet number of the main spike, and grain number per spike in 2017 (Table 2). The average main spikelet of later individuals (23.8) was significantly higher than that of slightly delayed heading individuals (20.3) and early individuals (19.8), while the average grain number of the main spike of later individuals (53.2) and slightly delayed heading individuals (40.1) was significantly higher than that of early individuals (22.0). There were no significant differences among early, slightly delayed, and later individuals in average plant height (87.8 cm, 86.3 cm, and 88.1 cm, respectively), average main spike length (11.2 cm, 11.4 cm, and 11.8 cm, respectively), and average number of tilters (7.2, 6.8, and 8.3, respectively).

**Figure 3:**
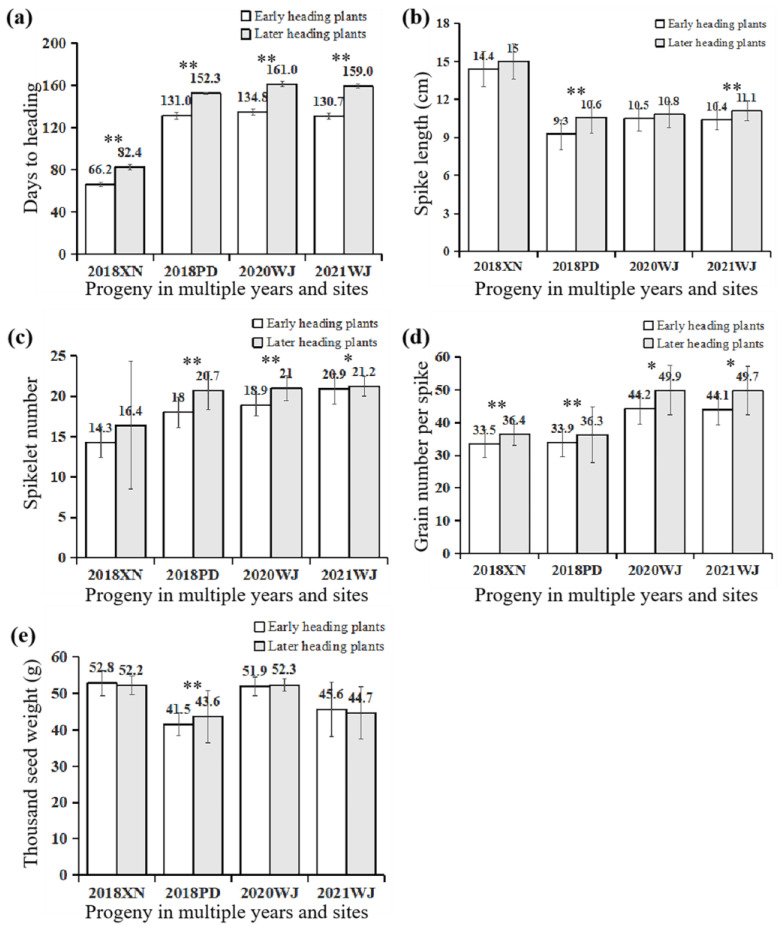
The trait values of stable progeny in multiple years and sites. (**a**) Days to heading of stable progeny in multiple years and sites; (**b**) Spike length (cm) of stable progeny in multiple years and sites; (**c**) Spikelet number of stable progeny in multiple years and sites; (**d**) Grain number per spike of stable progeny in multiple years and sites; (**e**) Thousand seed weight (g) of stable progeny in multiple years and sites. * and ** on the histogram suggest significance of the *t* test at *p* < 0.05 and *p* < 0.01, respectively.

Related traits of later individuals (more than 50) and early individuals (more than 45) were evaluated at multiple experimental stations in the following years (shown in Figure 3 for details). Later individuals were approximately 16 days later than early individuals at the spring-sown XN experimental station, while they were more than 20 days later, on average, at the remaining autumn-sown experimental stations. The average grain number per spike of later individuals was significantly (or significantly) higher than that of early individuals at all experimental stations, while the average main spikelet number of later individuals was greatly significantly (or significantly) higher than that of early individuals in autumn sown and not significantly higher than that of early individuals in XN. Furthermore, the thousand seed weights of later individuals were significantly heavier than or equal to those of early individuals. This indicates that the delay of heading can improve agronomic traits, especially grain number per spike, with no reduction in the thousand-seed weight.

### 2.3. Evaluation of the Different SNP Alleles between the Early Heading Pool and the Later Heading Pool

**Figure 4:**
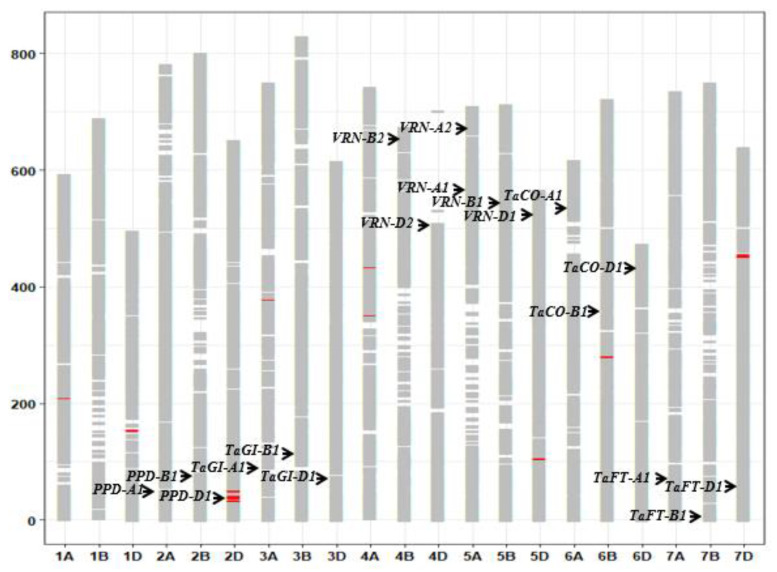
The different SNP alleles and graphical genotypes between the early heading pool and later heading pool. The red boxes refer to different segments. The black arrowheads indicate major genes for heading.

In all, 53,063 SNP sequences were mapped onto the Chinese Spring reference sequence: 17,879 mapped to A genome sites, 18,203 mapped to B genome sites, and 15,078 mapped to D genome sites. Except for chromosome 4D, which was marked by 1654 SNP loci, each chromosome had at least 2100 markers. On the basis of allelic status at the 53,063 SNP sites, eleven different segments on chromosomes 1A, 1D, 2D, 3A, 4A, 5D, 6B, and 7D were revealed between the early heading pool and later heading pool. One of them (32.8–42.9 Mb) contained the *PPD-D1* (TraesCS2D01G079600) region (Figure 4).

### 2.4. PPD-D1 Allele Detection

Because the photoperiod gene *PPD-D1* has a great influence on the regulation of wheat heading and flowering [20,21,28,29,30,31,36,37], molecular markers of *PPD-D1* for the 2 kb deletion assay upstream [32] and the developed KASP (Kompetitive Allele Specific PCR) molecular markers (named KASP587) for the 5 bp deletion assay in exon 7 were used to detect sequence variations.

**Figure 5:**
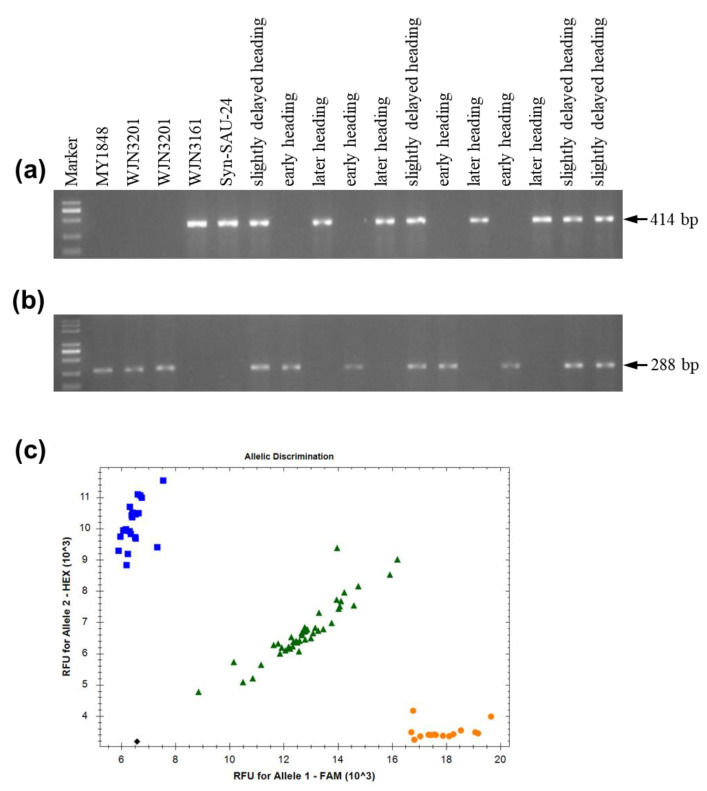
Molecular marker assay. (**a**) Ppd-D1_F/R1; (**b**) Ppd-D1_F/R2; (**c**) Exon 7 KASP markers. Blue square, yellow circle, and green triangle represent intact exon 7, 5 bp deletion in exon 7, and heterozygous genotype, respectively.

In the promoter region, if the amplification using primers Ppd-D1_F and Ppd-D1_R1 produced a 414 or 453 bp band, the variety carried the recessive day-length sensitive allele *ppd-D1b*, a 288 bp fragment was amplified using primers Ppd-D1_F and Ppd-D1_R2, and the variety carried the dominant insensitive allele *Ppd-D1a* [15]. Using the Ppd-D1_F and Ppd-D1_R1 primers, Syn-SAU-24 and WJN3161 (later heading date) amplified a 414 bp fragment, which differed from those produced by WJN3201 (early heading date) with no amplicon (Figure 5a). Meanwhile, using the Ppd-D1_F and Ppd-D1_R2 primers, WJN3201 (early heading date) amplified a 288 bp fragment, which differed from those produced by Syn-SAU-24 and WJN3161 (later heading date) with no amplicon (Figure 5b). Further analysis of two F_7_ segregation populations from WJN3141 and WJN3151 revealed early heading plants, slightly delayed heading plants, and later heading plants, which amplified a no amplicon, 414 bp fragment, and a 414 bp fragment by the primers Ppd-D1_F and Ppd-D1_R1, respectively (Figure 5a). Moreover, early heading plants, slightly delayed heading plants, and later heading plants amplified a 288 bp fragment, a 288 bp fragment, and a no amplicon by the primers Ppd-D1_F and Ppd-D1_R2, respectively (Figure 5b). Using the developed KASP markers, Syn-SAU-24 and early heading plants carried homozygous intact exon 7, while later heading plants carried the homozygous 5 bp deletion in exon 7, and slightly delayed heading plants carried the heterozygous genotype in exon 7 (Figure 5c). The summary of all molecular analysis and the genotypes of plant materials are shown in Table 3. Resequencing of the *PPD-D1* gene showed that *PPD-D1* of Syn-SAU-24 (accession no. MZ821039) indeed carried intact exon 7.

The results indicate that delays in heading are associated with *PPD-D1*, while the 2 kb upstream deletion or 5 bp deletion in exon 7 is responsible for heading date segregation.

### 2.5. Association of 2 kb Insertion with Later Heading

In *PPD-D1*, photoperiod insensitivity is associated with a candidate 2kp deletion upstream of the coding region [32]. Moreover, the 5 bp deletion in exon 7 created a frame shift and resulted in a non-functional protein [15]. Based on a preliminary wheat screen, we found that the common wheat line MY1848 (with a 2 kb upstream deletion) had no band and a 288 bp fragment in two polymorphic sites of the promoter region and carried a 5 bp deletion in exon 7 by marker analysis (Figure 5). The resequenced *PPD-D1* of MY1848 has also been deposited in GenBank as MZ821039. The homozygous later heading individuals 18DTN45 from WJN3161 (with the 2089 bp intact region and 5 bp deletion in exon 7) were then crossed with MY1848 to develop F_1_, F_2_, and F_2:3_ populations for genetic analysis of the association of the 2 kb insertion with later heading.

**Figure 6:**
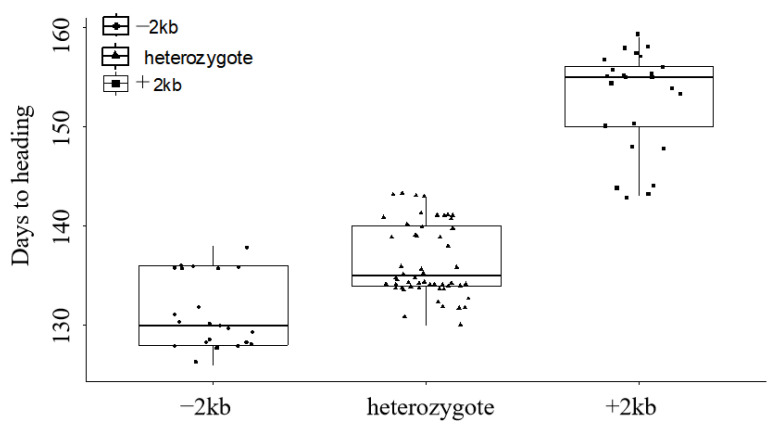
The frequency distribution of days to heading in the MY1848/18DNT45 F_2_ population.

MY1848 had an early heading date, with 125 days from sowing to heading, and 18DTN45 had a late heading date, with 150 days from sowing to heading. F_1_ plants exhibited 132 days to heading, slightly delayed compared with MY1848. According to the results of the heading date analysis of the F_2_ and F_2:3_ populations, the F_2_ population showed a segregation of 24 (homozygous early heading ranged from 126 to 139 days, average 131 days): 54 (heterozygous ranged from 130 to 143 days, average 137 days): 25 (homozygous later heading ranged from 141 to 159 days, average 153 days), fitting a 1:2:1 ratio (χ^2^_1:2:1_ = 0.262, *p* = 0.877) (Figure 6).

**Figure 7:**
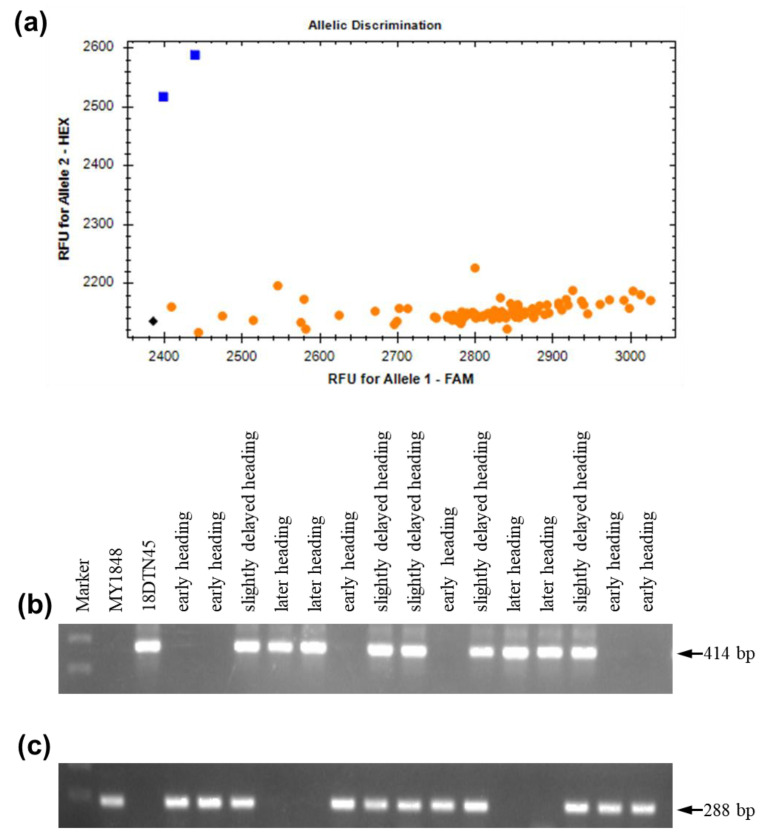
Molecular marker analysis of the MY1848/18DNT45 F_2_ population. (**a**) KASP markers for the 5 bp deletion assay in exon; (**b**) Ppd-D1_F/R1; (**c**) Ppd-D1_F/R2. The blue square (positive control) and yellow circle represent intact exon 7 and a 5 bp deletion in the exon 7 genotype, respectively.

All plants from the F_2_ and F_2:3_ populations for genetic analysis of the association of the 2 kb insertion with later heading had a 5 bp deletion in exon 7 (Figure 7a). Using the 2 kb deletion markers for further analysis, early heading homozygotes had no band and 288 bp fragment in two polymorphic sites, while later heading homozygotes had 414 bp amplification and no amplification in two polymorphic sites (Figure 7b,c). Meanwhile, heterozygotes had 414 bp amplification and 288 bp amplification in two polymorphic sites (Figure 7b,c). These findings confirmed that the 2 kb insertion was associated with delays in heading.

## 3. Discussion

### 3.1. A 2 kb Insertion in the Sensitivity PPD-D1 Allele Is Responsible for Delays in Heading with Improvement in Agronomic Traits

As a widely cultivated crop, wheat has formed diverse ecological types adapted to different conditions worldwide [23]. Furthermore, bread wheat is cultivated in virtually all countries where the photoperiod varies dramatically and continually, suggesting that there may be corresponding variations in photoperiod response among wheat varieties adapted to different environments [15]. The *Ppd-1* genes confer photoperiod insensitivity, permitting early flowering and are considered to have pleiotropic effects on other agronomic traits, reducing plant height, tillering, and spikelet numbers and increasing spikelet fertility [22]. The most intense genetic effect is exerted by the dominant *PPD-D1 allele*, followed by the dominant *PPD-B1* and *PPD-A1* alleles [20,21,28,29,30,31,36,37].

The *PPD-D1* locus was shown to control photoperiod-dependent floral induction and to have a major inhibitory effect on paired spikelet formation by regulating the expression of *FT* [16]. *Ppd-1*-insensitive alleles decrease the maximum number of florets initiated by shortening the floret initiation phase, which results in a lower number of fertile florets at anthesis [30]. Varieties with the photoperiod insensitive *Ppd-D1a* allele, which causes early flowering in short days or long days, had a 2 kb deletion upstream of the coding region, and the candidate mutation of the 2 kb deletion was considered associated [32]. Moreover, the 5 bp deletion in exon 7 created a frame shift and resulted in a non-functional protein [15,38,39].

In this study, near-isogenic lines derived from synthetic wheat showed a wide range of heading date and spike characteristics and were used to understand the *PPD-D1* gene responsible for the delay of heading with agronomic traits. Our results showed that heading time segregation was conferred by a single incomplete dominant *PPD-D1* gene, and the haplotype of the *PPD-D1* gene consisted of a 2 kb insertion of Syn-SAU-24 in the promoter region and a 5 bp deletion in exon 7, resulting in delayed heading time compared with the insensitivity allele. Further analyses indicate that the 2 kb insertion in the promoter region is responsible for the delay of heading (Figure 6 and Figure 7). Meanwhile, on the basis of resequencing *Ppd-D1* of MY1848 (accession no. MZ821040) and amplification by molecular markers (Figure 5), the common wheat line MY1848 carried a new insensitive *Ppd-D1* haplotype, and their only difference was the 5 bp deletion in exon 7 compared with haplotype I identified by Guo [15]. In addition, slightly delayed heading plants had a significantly increased grain number of the main spike compared with early plants (Table 2), while later heading plants had a significantly increased grain number and spikelet number of the main spike compared with early plants at all experimental stations and autumn sowing stations (Table 2 and Figure 3). This indicates that the delay of heading showed breeding potential with improvement in agronomic traits.

### 3.2. Incomplete Dominant Gene PPD-D1 Gene Model for Heterosis

Increasing grain yield is a long-term goal in crop breeding to meet the demand for global food security. Heterosis occurs when a hybrid shows higher performance for a trait than both parents, making heterosis breeding a powerful way to meet global food security demands. Hybrid rice not only provides new methods for utilizing heterosis in other crops, but it also contributes to food security in China and the rest of the world [40]. In hybrid rice varieties, the heterozygous first filial (F_1_) generation displays a yield advantage of 10–20% over their inbred parental lines [41,42,43]. Heading date-related genes (such as *Ghd8*, *Ghd*7, *Hd1*, and *Hd3*) were previously reported to underlie yield heterosis in most cases by affecting traits [44,45,46,47,48,49,50]. *Hd3* (rice orthologue of the *FT* gene) is associated with heterosis and delay of flowering. Compared to the genotype in male parents (*Hd3a*/*Hd3a*), the heterozygous state (*Hd3a*/*hd3a*, only slightly delayed flowering time) showed an advantage of 7.4% in seed-setting rate and 9.9% in grain yield per plant; although the *hd3a*/*hd3a* genotype showed an increased grain yield, it also showed delayed rice flowering (extending the growth stage by up to 20%), which would be impractical for agricultural production in most rice planting areas. Hence, when considering both yield and timing, the performance of *Hd3a*/*hd3a* was better than that of either of the two homozygous genotypes [49]. In this study, homozygous genotypes (*Ppd-D1a*/*ppd-D1b*) showed approximately nine days of delayed heading and great improvement in grain number per spike compared with early plants (*Ppd-D1a*/*Ppd-D1a*) (Table 2). These findings reinforce the idea that utilization of the photoperiod sensitivity gene *ppd-D1b* is a potential means to increase wheat yield potential.

### 3.3. Implication for Breeding

In addition to improving yields by altering spike morphology, breeders have aimed to develop wheat varieties specifically adapted to different environments, thereby ensuring maximum production. The alleles, haplotypes, and copy number variation identified for *Vrn* and *Ppd* genes respond differently in different climatic conditions and thus could alter not only the development phases but also the yield [51]. Late onset and/or a slower rate of leaf senescence confers a yield advantage in several crops, particularly under water stress environments [52,53,54,55] and heat stress environments [56,57]. Stay-green types maintain photosynthetic ability and may be important for yield improvement. The positive correlation between the green leaf duration after heading (GLDAH) and grain volume and kernel weight found then stable QTL co-segregated with *PPD-D1* and *VRN-B1* impacted GLDAH were reported [58], Meanwhile, a positive correlation between GLDAH and test weight, seed weight, and seed diameter were found [58,59]. In addition, *Ppd-1* is a key regulator of inflorescence architecture and paired spikelet development in wheat [16]. It was shown that *Ppd-1a* decreases the maximum number of florets initiated by shortening the floret initiation phase [30]. Lengthening the preanthesis period of stem elongation by altering photoperiod sensitivity has been suggested as a potential means to increase the number of fertile florets at anthesis to increase wheat yield potential [30].

In this study, later heading plants (*ppd-D1b*/*ppd-D1b*) stayed green until harvest (Figure 1) and showed significantly increased grain number and spikelet number of the main spike compared with early plants at all experimental stations and autumn sown stations (Table 2 and Figure 3). We checked grain plumpness harvested from 2019 to 2021, and there were shrivelled seeds from early heading individuals and later heading individuals. We also found plump seeds with good annual repetition from later heading individuals. This indicates that the grain filling rate is not directly correlated with heading date. Grouting speed, later period, and ripe grain full later heading individuals showed breeding potential with improvement in agronomic traits.

## 4. Materials and Methods

### 4.1. Plant Materials

The plant materials used in this study included synthetic hexaploidy wheat Syn-SAU-24 (2n = 6x = 42, AABBDD) with later heading (bred from a chromosome-doubled amphiploid between *T. turgidum* accession AS2291 as the female parent and *Ae. tauschii* accession 2404 as the male parent), early heading common wheat lines Mian-yang1848 (MY1848), and four F_6_ derivatives (WJN3201 with early heading data, WJN3161 with later heading date, WJN3141 and WJN3151 with slightly delayed heading) derived from just one F_5_ plant through the double top-cross (SHW/backcross wheat 1//backcross wheat 2///backcross wheat 3) between Syn-SAU-24 and three common wheats (unknown) during the breeding population development. Four derivatives were generated by two-phase selection for desirable agronomic traits [14].

One progeny plant (18DTN45) from WJN3161 was crossed with MY1848 to develop F_1_, F_2_ and F_2:3_ populations as validation populations for the association of the 2 kb insertion/deletion in the promoter region from *PPD-D1* with heading date.

### 4.2. Field Trials and Trait Evaluation

Four F_6_ derivatives and parents were planted at the Wenjiang (WJ) Experimental Station (30.7° N, 103.9° E, ~525 m a.s.l) in a ten-row plot with a 2.0 m row length and 20 plants per row, with each row separated from its neighbour by 30 cm in the 2016–2017 cropping season.

The seeds of each individual from the heading date segregated F_6_/F_7_ derivatives and parents were planted at the Pidu (PD) experimental station (30.4° N, 103.4° E, ~513 m a.s.l) in 2017–2018, Xining (XN) experimental station (36.6° N, 101.8° E, ~2110 m a.s.l) in 2018, and WJ Experimental Station in 2019–2020 and 2020–2021. Each line was planted in a 2.0 m row length with 20 plants per row, with each row separated from its neighbour by 30 cm. Field management, including irrigation, fertilization, and harvesting date, was performed according to local practices.

Validation populations were also planted at the WJ Experimental Station in 2019–2020 and 2020–2021.

The heading date was calculated as days from the sowing date to the date when approximately 50% spikes were visible. At maturity, each plant of the heading date-segregated population from the F_6_ derivatives was measured for plant height (the tallest culm, awns excluded), tiller number, spike length, and spikelet number per main spike. Then, all spikes were harvested to calculate the grain number per spike. Ten other lines were selected in the middle rows to measure tiller number, spike length, and spikelet number per main spike. Then, 10 main spikes were harvested to calculate grain number and measured (after drying) kernel weight. The average value for each trait was then calculated. dates were analysed by Excel 2007 and SPSS 2.0.

### 4.3. SNP Genotyping

Genomic DNA was extracted from young, fresh leaves using a plant genomic DNA kit (DP305) from Tiangen Biotech Co. (Beijing, China). The genomic DNA of 15 extreme phenotype individuals from WJN3141 segregation populations was bulked in an equal ratio to generate the early heading bulked DNA pool and later heading bulked DNA pool separately. Chip-based genotyping was carried out using the Wheat 55 K SNP array containing 53,063 markers by CapitalBio Technology (Beijing, China) “https://www.capitalbio.com/ (accessed on 7th December 2018)”. Markers showing homozygous genotypes among the early heading pool, later heading pool, and Syn-SAU-24 were used to analyse the different segments between the early heading pool and later heading pool and possible Syn-SAU-24 donor segments in the two pools.

The ratios of the same SNP to the total SNPs scored between the early heading pool and later heading pool were calculated using a sliding window of 10 Mb and step length of 1 Mb as described by Hao et al. [14]. Only results from windows with >30 markers were treated as effective data. The different genome regions of two pools covered by windows with a higher ratio of the same SNP to Syn-SAU-24 larger than 0.6 were defined as possible Syn-SAU-24 donor segments. Graphical representations were constructed using the R package ggplot2 (v.2.2.1) [60].

### 4.4. DNA Amplification and Sequencing

We used polymorphic site markers to identify each *PPD-D1* genotype detailed in Table 4: a common forwards primer Ppd-D1_F combined with two reverse primers, Ppd-D1_R1 and Ppd-D1_R2, were used for the 2 kb deletion assay upstream [32]; Kompetitive Allele Specific PCR KASP markers (Exon 7_KASP587-1F and Exon 7_KASP587-2F combined with Exon 7_KASP587-R) were developed to detect the 5 bp deletion in exon 7 by DNAMAN version 8.0 (Lynnon Biosoft, Quebec, QC, Canada). The genotyping reactions were set up using a CFX96 Touch Real-Time PCR Detection System (Bio-Rad, Hercules, CA, USA) in a final volume of 10.4 µL with 1 ng genomic DNA, 5 µL KASP reaction mix, 1.4 µL primer mix, and 3 µL nuclease-free water. Polymerase chain reaction (PCR) conditions were set as 10 min at 95 °C, 10 touchdown cycles of 20 s at 95 °C, 1 min at 65–57 °C (dropping 1 °C per cycle), and 35 cycles of 20 s at 95 °C, 40 s at 55 °C. Fluorescence detection of the reactions was performed using a Bio-Rad CFX manager 3.1 (Bio-Rad, USA).

The differences in the sequences of *PPD-A1*, *PPD-B1*, and *PPD-D1* from GenBank (NCBI) were also used to design the D genome-specific primers. The *PPD-D1* gene sequence was amplified as three separate overlapping fragments using three primers, Ppd-D1_Frag1F/Ppd-D1_Frag1R, Ppd-D1_Frag2F/Ppd-D1_Frag2R, and Ppd-D1_Frag3F/Ppd-D1_Frag3R (detailed in Table 4).

The PCR amplifications were performed using a PCR-200 Thermocycler (MJ Research, Watertown, MA, USA). Amplification of the DNA was performed in a volume of 50 µL, which contained 200 ng template DNA, 200 µmol/L each dNTP, 100 µmol/L each primer, 5.0 µL of 10× PCR buffer, 1 U ExTaq DNA polymerase with high fidelity (TaKaRa, Dalian, China), and double-distilled (dd) H_2_O. A detailed description of the reactions is shown in Table 4. The amplified products were separated on 1.0–3.0% (amplicon-dependent) agarose in 1×TAE buffer (0.04 mol/L Tris base, 0.02 mol/L acetic acid, and 1.0 mmol/L EDTA), followed by staining with ethidium bromide.

The PCR products were purified from agarose gels using an E.Z.N.A. Gel Extraction Kit (Omega Biotek, Doraville, GA USA) to obtain the desired amplicons. Purified products were ligated into 0.5 ng PMD18-T vectors (TaKaRa) and transformed into ES-cherichia coli DH5α-competent cells. Cells were plated onto LB agar-ampicillin plates in the presence of 40 µL of 20 mg/mL 5-bromo-4-chloro-3-indolyl β-d-galactoside (X-gal) and 4 µL of 200 mg/mL isopropyl β-D-thiogalactopyran-oside (IPTG), allowing blue-white selection, and grown overnight at 37 °C. Five to 10 white candidate colonies from each plate were then grown overnight at 37 °C on LB agar-ampicillin plates, and positive clones were identified by PCR primers for confirmation. Positive clones for each fragment were forwards and reverse sequenced by SunBiotech (Beijing, China). Complete sequences were assembled using the Sequence Assembly Editor of DNAMAN version 8.0 (Lynnon Biosoft, Quebec, Canada).

## Figures and Tables

**Table 1:** Analysis and description of heading date in segregation populations.

Populations	No.	Heading Date			Expected Ratio	χ2	*p*-Value
		Early	Segregation	Later			
WJN3141_F_7_	150	121 (≤153 days)		29 (≥159 days)	3:1	2.569	0.109
WJN3141_F_7:8_	150	42 (≤143 days)	79	29 (≥159 days)	1:2:1	2.680	0.262
WJN3151_F_7_	151	118 (≤153 days)		33 (≥162 days)	3:1	0.797	0.372
WJN3151_F_7:8_	151	31 (≤141 days)	87	33 (≥162 days)	1:2:1	3.556	0.169

**Table 2:** Measurement and description of traits in two F_7_ segregation populations.

Code	No.	Days to Heading	Plant Height (cm)	Tiller No.	Main Spike Length (cm)	Spikelet Number of Main Spike	Grain Number per Spike
early heading plants	73	135.9 ± 2.1	87.8 ± 3.9	7.2 ± 2.4	11.2 ± 1.0	19.8 ± 1.2	22.0 ± 4.5
slightly delayed heading plants	166	145.0 ± 2.3 **	86.3 ± 9.6	6.8 ± 2.6	11.4 ± 6.7	20.3 ± 1.8	40.1 ± 3.5 **
later heading plants	62	162.7 ± 2.7 **	88.1 ± 5.9	8.3 ± 2.5	11.8 ± 1.3	23.8 ± 1.8 **	53.2 ± 5.2 **

**, significantly different from early heading plants at *p* = 0.01, respectively (*t* test).

**Table 3:** The summary of all molecular analysis and the genotypes.

Code	Phenotypes	Polymorphic Site Markers			Genotypes
		Ppd-D1_F/R1	Ppd-D1_F/R2	Exon 7_KASP587	
Syn-SAU-24	later heading	414 bp	–	intact	*ppd-1b*
MY1848	early heading	–	288 bp	5 bp deletion	*Ppd-1a*
WJN3201	early heading	–	288 bp	intact	*Ppd-1a*
WJN3161	later heading	414 bp	–	5 bp deletion	*ppd-1b*
18DTN45	later heading	414 bp	–	5 bp deletion	*ppd-1b*
WJN3141	slightly delayed heading	414 bp	288 bp	intact/5 bp deletion	*Ppd-1a/ppd-1b*
WJN3151	slightly delayed heading	414 bp	288 bp	intact/5 bp deletion	*Ppd-1a/ppd-1b*

**Table 4:** PCR primer sequences used for the amplification of Ppd-D1.

Target	Primer Name	Sequence of Primer (5′-3′)	Initial Denaturation	35 Cycle	Final Extension	Size (bp)
in the promoter region	Ppd-D1_F	ACGCCTCCCACTACACTG	94 °C/5 min	94 °C/30 s, 54 °C/30 s. 72 °C/45 s	72 °C/10 min	414 or 453 bp/no amplification
	Ppd-D1_R1	GTTGGTTCAAACAGAGAGC				
	Ppd-D1_F	ACGCCTCCCACTACACTG	94 °C/5 min	94 °C/30 s, 54 °C/30 s. 72 °C/2.5 min	72 °C/10 min	288 bp/no amplification
	Ppd-D1_R2	CACTGGTGGTAGCTGAGATT				
5 bp deletion in exon 7	Exon 7_KASP587-1F	GAAGGTGACCAAGTTCATGCTAATCAAGGCGGTGCAGGGTTC	/	/	/	/
	Exon 7_KASP587-2F	GAAGGTCGGAGTCAACGGATTAATCAAGGCGGTGCAGGGTTG				
	Exon 7_KASP587-R	TTGCTTCATCTGAGCGGCGTC				
5’UTR to 3’UTR	Ppd-D1_Frag1_F	GGCCCACAAAATCCACATCC	94 °C/5 min	94 °C/30 s, 59 °C/30 s. 72 °C/2.5 min	72 °C/10 min	1829 bp
	Ppd-D1_Frag1_R	ATTGGAATCATCGCCACTCT				
	Ppd-D1_Frag2_F	AGAGTGGCGATGATTCCAAT	94 °C/5 min	94 °C/30 s, 59 °C/30 s. 72 °C/40 s	72 °C/10 min	416 bp
	Ppd-D1_Frag2_R	TGGACAAATTGACCTCTAGTGCA				
	Ppd-D1_Frag3_F	TGCACTAGAGGTCAATTTGTCCA	94 °C/5 min	94 °C/30 s, 59 °C/30 s. 72 °C/3 min	72 °C/10 min	2766 bp
	Ppd-D1_Frag3_R	GCGGAATGAATTGCGCTTTCA				

## Data Availability

The data presented in this study are available in this article.
